# Genome-wide expression analysis offers new insights into the origin and evolution of *Physcomitrella patens* stress response

**DOI:** 10.1038/srep17434

**Published:** 2015-11-30

**Authors:** Basel Khraiwesh, Enas Qudeimat, Manjula Thimma, Amphun Chaiboonchoe, Kenan Jijakli, Amnah Alzahmi, Marc Arnoux, Kourosh Salehi-Ashtiani

**Affiliations:** 1 Laboratory of Algal, Systems, and Synthetic Biology, Division of Science and Math, New York University Abu Dhabi, Abu Dhabi, UAE; 2 Center for Genomics and Systems Biology, New York University Abu Dhabi, Abu Dhabi, UAE; 3 Division of Biological and Environmental Sciences and Engineering, King Abdullah University of Science and Technology, Thuwal, KSA

## Abstract

Changes in the environment, such as those caused by climate change, can exert stress on plant growth, diversity and ultimately global food security. Thus, focused efforts to fully understand plant response to stress are urgently needed in order to develop strategies to cope with the effects of climate change. Because *Physcomitrella patens* holds a key evolutionary position bridging the gap between green algae and higher plants, and because it exhibits a well-developed stress tolerance, it is an excellent model for such exploration. Here, we have used *Physcomitrella patens* to study genome-wide responses to abiotic stress through transcriptomic analysis by a high-throughput sequencing platform. We report a comprehensive analysis of transcriptome dynamics, defining profiles of elicited gene regulation responses to abiotic stress-associated hormone Abscisic Acid (ABA), cold, drought, and salt treatments. We identified more than 20,000 genes expressed under each aforementioned stress treatments, of which 9,668 display differential expression in response to stress. The comparison of *Physcomitrella patens* stress regulated genes with unicellular algae, vascular and flowering plants revealed genomic delineation concomitant with the evolutionary movement to land, including a general gene family complexity and loss of genes associated with different functional groups.

The moss *Physcomitrella patens* (*P. patens*) represents an important model plant species for understanding evolutionary dynamics of genes involved in different cellular processes^1^,^2^. Because mosses represent an evolutionary stage linking green algae to vascular plants, studies on extant mosses can yield insights on the evolution of plant responses to various environmental challenges and perturbations. Plant growth and yield potential are significantly influenced by various abiotic stresses caused by limited water availability on land, strong irradiation by sunlight, and varying temperatures. These stresses necessitate extensive modifications in signaling and physiological processes to modify body plans and activate complex regulatory networks^3^, which consist of transcriptional as well as post-transcriptional regulators^4^,^5^. Although the importance of abiotic stress signaling is recognized, some molecular components remain unknown. Additional, in-depth studies are needed to understand the species’ response to environmental factors limiting growth and development under non-ideal conditions.

The abiotic stress-associated hormone, Abscisic Acid (ABA) is a major player in mediating the adaptation of plants to stress and functions in many plant developmental processes^6^. For instance, ABA is required for the induction of genes as a response to dehydration stress^7^,^8^. While the transcriptional regulation of ABA response pathways has been intensively studied in seed plants^6^,^8^, the knowledge on ABA mediated gene expression in *P. patens* is currently restricted to the conserved ABA-mediated activation of ABA-responsive *cis*-elements (ABRE)^9^. Freezing or exposure to extremely low temperature constitutes a key factor influencing plant growth. Freezing stress at temperatures below 0 °C and chilling stress at non-freezing temperatures by which chilling tolerance is induced in plants is known as cold acclimation^10^. Sun *et al.*^11^ and Beike *et al.*^12^ have shown that cold acclimation in *P. patens* displays distinct differences in some aspects when compared to higher plants, suggesting significant alterations during the evolution of land plants.

Dehydration tolerance is an adaptive trait thought to have been necessary for the colonization of land by plants, and remains widespread among bryophytes^13^. *P. patens* has been classified as a drought tolerant plant since it has a high ability to recover water loss, even after a water loss of 92% on a fresh-weight basis^14^. *P. patens* has also been classified as a halophyte; a plant that naturally grows under high salt concentrations^15^. It can maintain growth at Na^+^ concentrations that would be detrimental to most vascular plants^14^,^16^,^17^.

However, in the field, plants are subjected to a combination of different stresses, e.g., drought often coincides with an increase in salinity or high temperature. Therefore, focus on molecular, physiological or metabolic research should not be limited to the effect of a single stress factor.

Based on EST-derived transcriptome analysis, the first microarrays were designed and used for comparative transcriptome analyses in *P. patens*, focusing on the identification of genes that respond to osmotic stress and ABA^9^,^14^,^18^. The transcriptome analysis was based on EST sequences derived mainly from partially sequenced cDNAs that are often incomplete in coding sequences and lack information from transcripts not represented in the RNA pools. High-throughput sequencing technologies are tremendously useful for analyzing transcriptome complexity and gene regulation. The RNAseq approach^19^ produces millions of short cDNA reads that are mapped to a reference genome to obtain a genome-wide transcriptional map that provides greater insight and accuracy than microarrays^19^,^20^,^21^,^22^. Here we used high-throughput Illumina HiSeq sequencing system for transcriptome analysis of *P. patens* under abiotic stress treatments. This identified temporal- and stress-specific candidate genes. Phylogenetic analyses of these genes in turn elucidated lineage-specificity of stress responses. The genes of the different expression clusters associated with different functional categories clearly indicate the biological, molecular and cellular events involved in *P. patens* stress responses.

## Results

### Transcriptome sequencing and mapping of the reads to *P. patens* reference genome

The annotated *P. patens* genome allowed analysis, identification and characterization of bryophyte genes^1^,^2^. For RNAseq, total RNA pooled from three biological replicates for each sample was subjected to cDNA library preparation to generate a broad survey of transcripts associated with the *P. patens* stress treatments. Raw Illumina sequencing reads were quality and adapter trimmed to yield a total of 220,031,432 short reads comprising 22.64 Gb of sequence data from the runs. Sequence reads were mapped to the *P. patens* genome annotation V1.6, which is 480 Mb in size with 32,272 protein-coding loci and 38,357 protein coding transcripts annotated^2^. Approximately 198.4 million reads, 89.79% of total reads, were perfectly aligned to the reference genome, and 91% of those matched annotated gene regions (Supplementary Table S1). Reads mapped to the annotated gene regions were counted to calculate RPKM to estimate gene expression^19^. Among the mapped reads, we found nearly 9.5 and 0.11 million paired reads located in the annotated exonic regions and across the exon-intron junctions, respectively.

### Global analysis of gene expression

**Figure 1:**
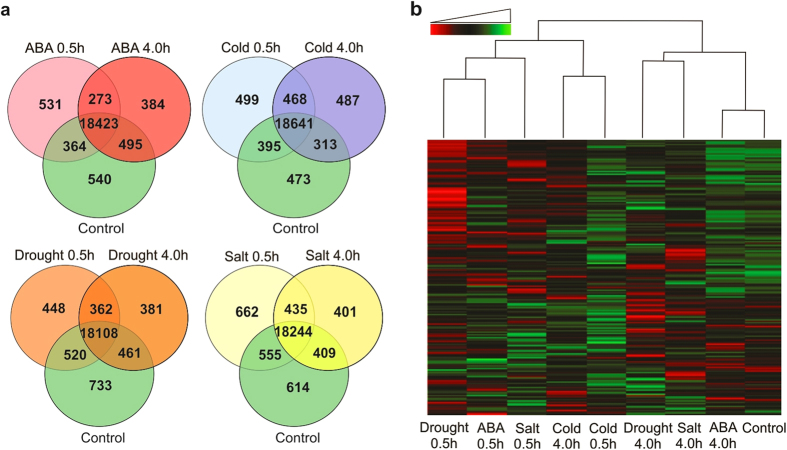
Global analysis of *P. patens* genes expression in response to abiotic stresses. A comparison of commonly and uniquely expressed genes across the treatments was done using the obtained RPKM values. The comparison was done for control versus all stress treatments including the indicated two time points. (**a**) Venn diagrams showing overlap of expressed genes relative to control among different subgroups of *P. patens* abiotic stress treatments. (**b**) Hierarchical clustering analysis and heatmap of gene expression based on log_2_ ratio RPKM data across abiotic stress treatments and the control sample. Red color represents lower expression, green color represents higher expression (the expression range from −5.644 to 12.889). The heatmap is based on the distance function 1, correlation between each test statistic of the expression of each gene, columns represent individual experiments, and rows represent transcriptional units. The dendrogram at the top indicates clusters of individual treatments.

One of the primary goals of RNA sequencing is to compare gene expression levels between samples. RNAseq data were processed to calculate RPKM values, a normalized measure of read density that allows transcript levels to be compared both within and between samples. A total of 23,971 genes were detected in the samples. Their expression in the four abiotic stress treatments with selected time points (0.5 and 4.0 h) are summarized in Fig. 1a. Venn diagrams show the distribution of expressed genes from abiotic stress treatments (ABA, cold, drought and salt) with selected time points (0.5 and 4.0 h) compared to the control sample (Fig. 1a, Supplementary Datasets 1–4). Among these genes, the number of stress-specifically expressed genes was 531 (ABA 0.5 h), 384 (ABA 4.0 h), 273 (ABA 0.5/4.0 h), 499 (cold 0.5 h), 487 (cold 4.0 h), 468 (cold 0.5/4.0 h), 448 (drought 0.5 h), 381 (drought 4.0 h), 362 (drought 0.5/4.0 h), 662 (salt 0.5 h), 401 (salt 4.0 h), and 435 (salt 0.5/4.0 h). Although there were 17,381 genes expressed among all stress treatments and control sample, many of them were quantitatively regulated (Fig. 1b, Supplementary Dataset 5); while some of these genes had little variation across abiotic stress treatments, suggesting they fulfill housekeeping functions.

Hierarchical clustering was performed using Pearson’s correlation coefficient as a distance metric and using the average agglomeration method^23^. The genes were clustered according to the expression profile in the samples. The clustering shows that the samples of ABA 4.0 h and control sample have a high correlation of 0.91 indicating that the variables are positively and linearly related between these samples. Moreover, other stress treatment samples have a correlation coefficient of 0.73 with the control, and the variables were less related for these treatments. Generally, early and late response time points did not cluster together, with the exception of cold treatment, where both the 0.5 and 4 h time points were found to cluster together (Fig. 1b). To investigate if this consequence of a specific clustering strategy that was used, different clustering methods were used based on either correlation or Euclidean distance with complete and average linkage, the dendrograms show the clustering of the expression profiles of stress treatments and the control sample (Supplementary Fig. S1). The results show that control and ABA 4.0 h tend to cluster together and salt 4.0 h and drought 4.0 h tend to cluster together while the rest of stress treatments closely grouped (Supplementary Fig. S1). Additionally, Principal Component Analysis (PCA) was preformed in order to summarize the systematic patterns of variations in the data and the grouping of expression profiles of each sample in the experiment. In agreement with clustering analysis, the PCA shows that control and ABA 4.0 h expression profiles closely cluster, salt 4.0 h and drought 4.0 h also have similar profiles, and the expression profiles of cold 0.5 h and 4.0 h treatments tend to cluster with ABA 0.5 h, drought 0.5 h and salt 0.5 h (Supplementary Fig. S2).

### Changes in gene expression profiles of different abiotic stress treatments

**Figure 2:**
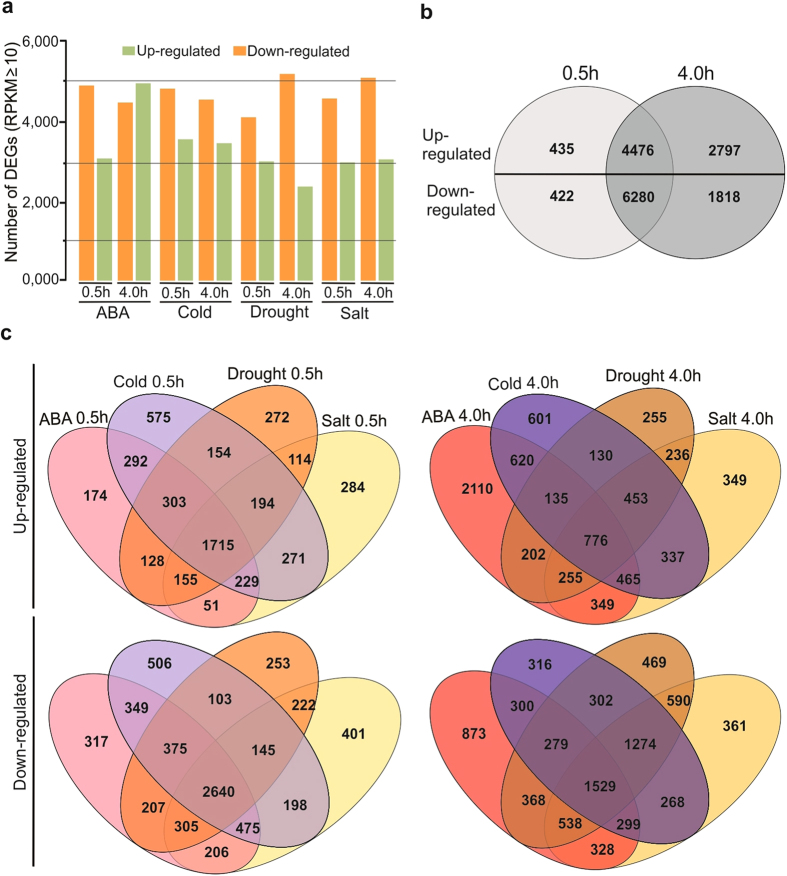
Differential expression of *P. patens* genes in response to abiotic stresses. Differentially Expressed Genes (DEGs) were identified relative to the control sample grown under standard conditions. The differences in gene expression among the abiotic stress treatments and the control sample were obtained based on the RPKM-derived read count using a Log_2_ Ratio calculation. (**a**) Number of up regulated (green bars) and down regulated (orange bars) genes are shown for each stress treatment at the time point in hours. (**b**) Venn diagram showing overlap of DEGs among the two time points (0.5 and 4.0 h). (**c)** Venn diagrams showing overlap of DEGs in response to the four assayed abiotic stresses at two time points (0.5 and 4.0 h). Upper diagrams indicate up regulated genes and lower ones indicate down regulated genes. The numbers of genes in each region of the diagrams are indicated. The Venn diagrams depict the overlaps between each pairwise comparison.

Differentially Expressed Genes (DEGs) were identified relative to the control sample grown under standard conditions (see Methods). To obtain significant differences in gene expression among the abiotic stress treatments and the control sample, we compared the RPKM-derived read count using a Log_2_ Ratio calculation (a log ratio of 1 represents a 2-fold change). There were about 17,381 genes that were expressed across all stress conditions with RPKM values above zero. To minimize false positives, we set a relatively conservative threshold of an RPKM value ≥10. The results indicated that a set of 7,921 (ABA 0.5 h), 9,426 (ABA 4.0 h), 8,524 (cold 0.5 h), 8,084 (cold 4.0 h), 7,285 (drought 0.5 h), 7,791 (drought 4.0 h), 7,605 (salt 0.5 h), 8,407 (salt 4.0 h) and 9,668 genes across all stress treatments without any redundancy were DEGs above RPKM 10 (Fig. 2a, Supplementary Dataset 6).

To identify genes showing a significant change in expression among the two time points (0.5 and 4.0 h) we compared all DEGs across all stress treatments. A total of 12,184 genes were up regulated and 14,800 genes were down regulated; the number of DEGs uniquely appearing in 0.5 h time point was 4,911 (up regulated) and 6,702 (down regulated), while 7,273 (up regulated) and 8,098 (down regulated) genes were uniquely appearing in the 4.0 h time point (Fig. 2b, Supplementary Dataset 7). These results suggest that the differentiation of expressed genes are exposure-time dependent and they are more differentially expressed when the *P. patens* protonema tissues are exposed to the stress treatments for 4.0 h as compared to 0.5 h.

For all comparisons of pairs, a large number of DEGs were specific for a certain stress treatment and time point (Fig. 2c, Supplementary Datasets 8–11). Among all DEGs across all stress treatments for 0.5 h, transcripts of 1,715 genes were induced, and 2,640 were repressed persistently (Fig. 2c). Comparison of all DEGs across all stress treatments for 4.0 h showed transcripts of 776 genes were induced, and 1,529 were repressed persistently among all stress treatments (Fig. 2c). All significantly induced and repressed genes derived from pairwise comparisons were compared with each other, the Venn diagrams depict the overlaps between each pairwise comparison.

### Gene ontology and gene set enrichment analysis for DEGs

To facilitate more analysis on gene expression, all predicted *P. patens* stressed-DEGs were assigned to different functional categories using Blast2GO. The annotations were verified manually and integrated using gene ontology (GO) classification. For all DEGs, GO term annotations were analyzed; 8,383 of the 9,668 DEGs were annotated in at least one of the three GO categories: cellular component, biological process or molecular function; whereas 1,285 (13.29%) of the DEGs had no available GO annotation (Supplementary Datasets 6, 12–14). Several hundreds of genes related to translation, oxidation-reduction process, regulation of transcription, response to stress, protein binding, and ATP binding were enriched amongst differentially expressed genes in all stress treatments (Supplementary Fig. S3). Some of these functional groups were enriched with a large number of genes; metabolic process included 3,887 genes and was a dominant group under the main category of biological process for instance. Binding and cell functional groups comprised 4,121 and 6,214 genes, and were dominant in the main categories of molecular function and cellular component, respectively (Supplementary Datasets 12–14). We also detected a large number of genes in the functional groups of cellular process with 3,825 genes, catalytic activity with 3,466 genes, and cell part with 6,213 genes.

**Figure 3:**
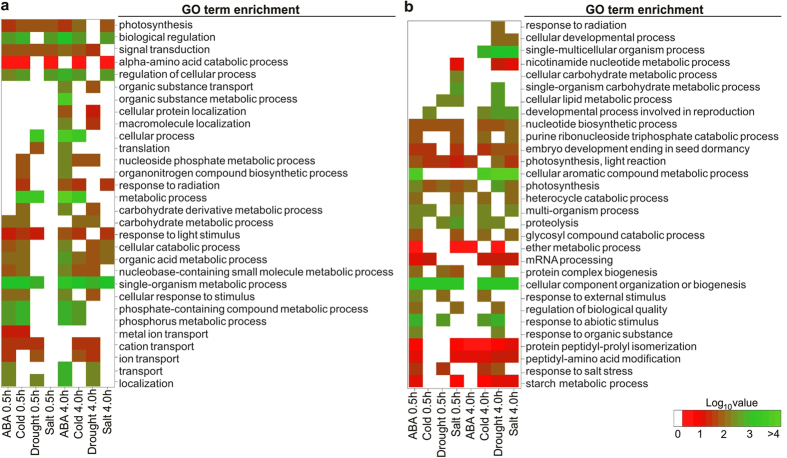
Heatmaps of gene set enrichment analysis (GSEA) of DEGs based on RNAseq data in response to abiotic stresses. GO annotation from *P. patens* genome annotation (v1.6) was analyzed with Blast2GO using the default parameters. The g:Profiler tool was used to classify the GSEA of *P. patens* stressed-DEGs for each condition based on up- and down-regulation status of the genes. (**a**) Selected functional groups for up regulated genes. (**b)** Selected functional groups for down regulated genes. The color intensities indicate the level of enrichment score of each GO term. Enrichments score is log_10_ (gene number). See Supplementary Datasets 15 and16 for the complete lists.

The g:Profiler tool^24^ was used to classify the gene set enrichment analyses (GSEA) of *P. patens* stressed-DEGs for each condition (ABA, cold, drought and salt) based on up and down regulated genes, and they were categorized into 710 and 579 functional groups, respectively (Supplementary Datasets 15 and 16). Among these groups, the terms related to biological process (GO:0008150), metabolic process (GO:0008152), cellular process (GO:0009987), biosynthetic process (GO:0009058), gene expression (GO:0010467), translation (GO:0006412) and response to stimulus (GO:0050896) were dominant in all stress treatments. Most of these groups showed statistically significant differences in GSEA based on GO terms (biological process) for up and down regulated genes (Fig. 3a,b). We detected a high percentage of down regulated genes from functional groups of single organism process (GO:0044699) in treatments of drought 0.5 h and cold 4.0 h, and high percentage of up regulated genes from functional groups of cellular metabolic process (GO:0044237) in ABA 4.0 h and cold 4.0 h treatments. Additionally, the up regulated genes from functional groups of signaling (GO:0023052) were present in ABA, cold and drought but not in salt treatments; and RNA metabolic process (GO:0016070) functional group for down regulated genes was present only in 4.0 h stress treatments. Interestingly, the functional group related to response to salt stress (GO:0009651) in down regulated genes was absent in salt 0.5 and 4 h (Fig. 3b). Furthermore, it was observed that some functional groups are present for certain stress treatments in up regulated genes yet were absent in the same treatments for down regulated ones and conversely. Notably, GSEA of DEGs was observed for different functional groups involved in different pathways, which suggests that there are considerable differences between the physiological processes among *P. patens* stressed-DEGs.

### The early stress response genes

Many genes display up or down regulation during early abiotic stress response (Fig. 2b). Identifying *P. patens* genes that are early regulators in stress responses and ABA signaling is critical for elucidating abiotic stress response mechanisms.

**Figure 4:**
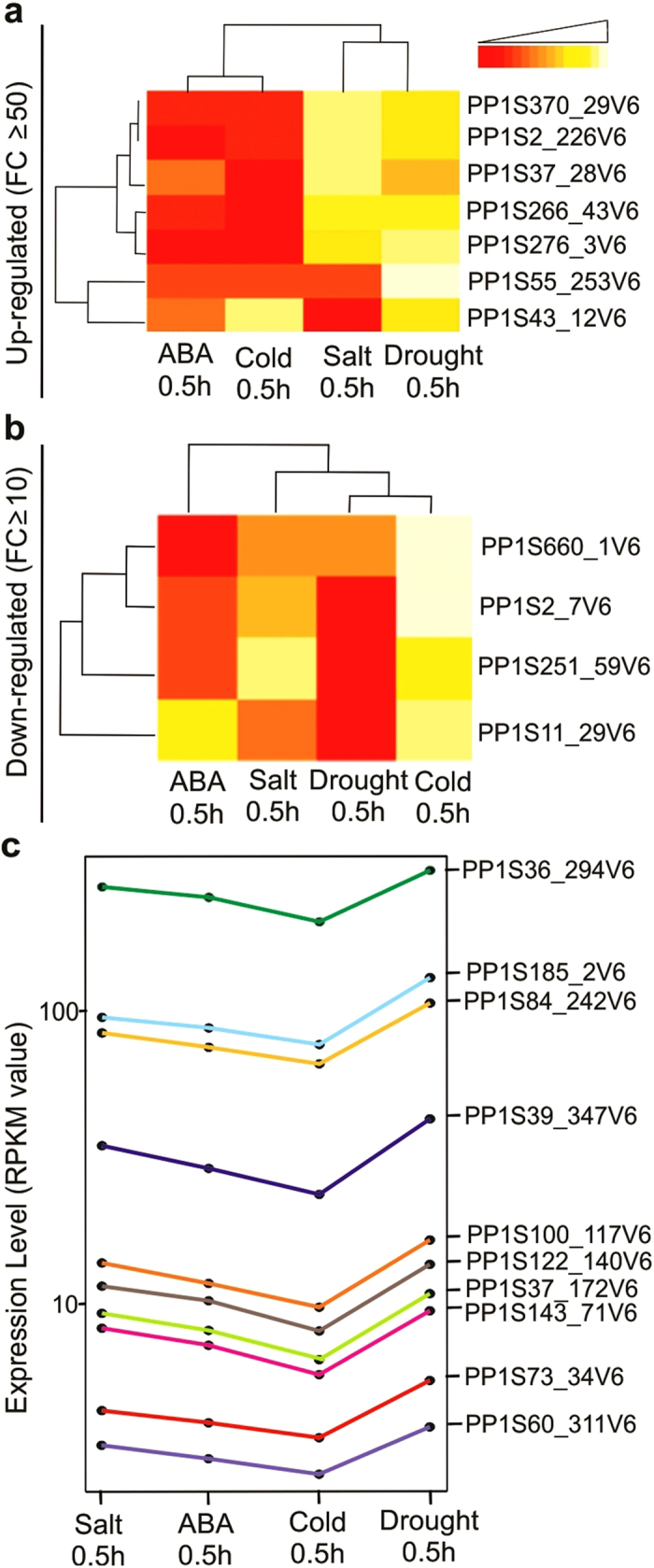
The early stress responses genes. Expression patterns of early ABA- and stress-responsive gene in *P. patens*, only genes with high fold change were considered. (**a**,**b**) Heatmaps comparison of gene expression among 0.5 h stress treatments with high fold change. Columns represent individual treatments, and rows represent transcriptional units. (**a)** Up regulated genes (**b**) Down regulated genes. (**c**), Group of early stress-expressed genes, they are in the same manner at low and high expression value.

To identify early genes strongly induced by stress treatments, we compared the RPKM-derived read count using a Log_2_ Ratio calculation to identify genes representing a high fold change in stress samples as compared to the control one at the 0.5 h time point. There were seven genes highly up regulated across all stress conditions at 0.5 h with ≥50 fold change (Fig. 4a). These highly regulated early stress genes have distinct functions (Supplementary Table S2). For example, Pp1s370_29V6.1 encodes late embryogenesis abundant protein, LEA-3. The broad subcellular distribution of LEA proteins highlights the need for each cellular compartment to be provided with protective mechanisms to cope with desiccation or cold stress, and this gene might be involved in maturation and desiccation tolerance of seeds also^25^. The Arabidopsis homolog of this transcript is involved in the salt stress pathway^26^. Pp1s43_3V6.1 and Pp1s55_253V6.1 are members of the AP2/EREBP family of transcription factors; recognize the drought-responsive element (DRE) in target promoters^27^. All of these genes appear to be over represented in salt and drought, where both of those treatments are clustered together, and less represented in cold and ABA treatments, where there is another cluster for those treatments (Fig. 4a). There were also four genes highly down regulated across all stress conditions at 0.5 h with a ≥10 fold change (Fig. 4b) and they encode different functions (Supplementary Table S3) such as beta-expansin 3 (Pp1s251_59V6.1), which has been shown to respond to water deficiency^28^, and another expansin transcript (Pp1s11_29V6.1), which is involved in plant-type cell wall organization and perhaps morphological adaptation.

Another group of early stress response genes were predicted at high and low expression levels based on absolute RPKM values (Fig. 4c, Supplementary Table S4). These co-expressed genes in early stress response might be lying in the same pathway. For example, Pp1s100_117V6.1 encodes ATP synthase beta subunit protein, which responds to oxidative stress. The acyl-CoA-binding protein 6 (Pp1s36_294V6.1) responds to both cold and salt stress^29^, and in agreement with our results, the expression of Pp1s36_294V6.1 increases in both early cold and salt stress (Fig. 4c). Other genes: Pp1s143_71V6.1, Pp1s37_172V6.1, Pp1s60_311V6.1, and Pp1s84_242V6.1 have no available GO annotation but they share the same expression profiles with the above transcripts. Interestingly, these genes show the same expression behavior among all stress treatments and appear to be expressed more in drought and less in cold than other treatments (Fig. 4c).

### Validation of *P. patens* gene expression patterns in response to abiotic stresses

**Figure 5:**
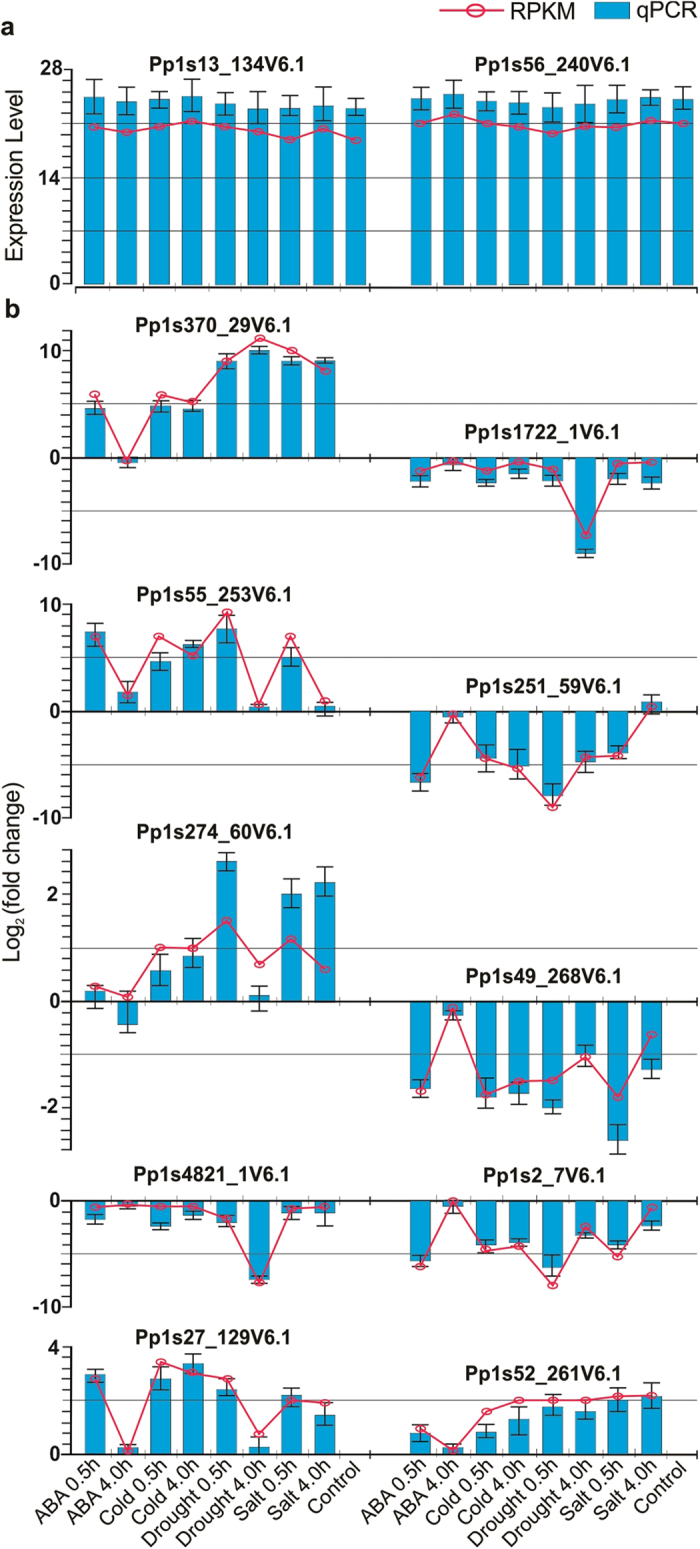
Validation of *P. patens* gene expression patterns in response to abiotic stresses. Selected expression gene profiles were validated with quantitative real-time PCR (qPCR). (**a**) qPCR validation and expression analysis of Pp1s13_134V6.1 and Pp1s56_240V6.1, which they were used as reference genes. (**b**) qPCR validation and expression analysis of 10 DEGs (up and down) under different stress conditions and they were encode different functions. Blue bars indicate the expression level and Log_2_ (fold changes) obtained from qPCR for the three replicates. Red lines indicate the expression level and Log_2_ (fold change) from RNAseq data analyses. Error bars represent the standard error of the mean.

We further validated the accuracy and reproducibility of gene expression results through using quantitative real-time PCR (qPCR) as an orthogonal method of gene expression analysis. The transcriptional levels of two reference genes and 10 DEGs were independently analyzed by qPCR (Fig. 5). Based on the RPKM results, the two genes (Pp1s13_134V6.1 and Pp1s56_240V6.1) are constitutively expressed with no significant difference among all stress treatments and control samples (Fig. 5a), and they encode 3-hydroxyisobutyryl-coenzyme A and riboflavin kinase, respectively. The qPCR results also showed the same expression patterns with no difference in expression among the samples tested (Fig. 5a and Supplementary Fig. 4). Thus, these two genes were considered endogenous controls (reference genes) for qPCR data normalization. Additionally, we designed primer pairs to specifically detect the transcript level of 10 DEGs (up- and down-regulated) that were encoding different functions (Supplementary Table S5). The transcript levels of the 10 DEGs were obtained by the qPCR assays, normalized with the above reference genes and compared with RPKM-derived read count using a Log_2_ Ratio calculation. Indeed, the transcript levels for the 10 selected genes were differentially regulated under stress conditions, and the expression patterns showed high degrees of concordance between qPCR assays and RNAseq analyzed data (Fig. 5b). Taken together, this independent qPCR evaluation confirms the reproducibility and validity of our method for identifying RNAseq-derived expression patterns.

### Evolutionary conservation of stress-regulated genes

**Figure 6:**
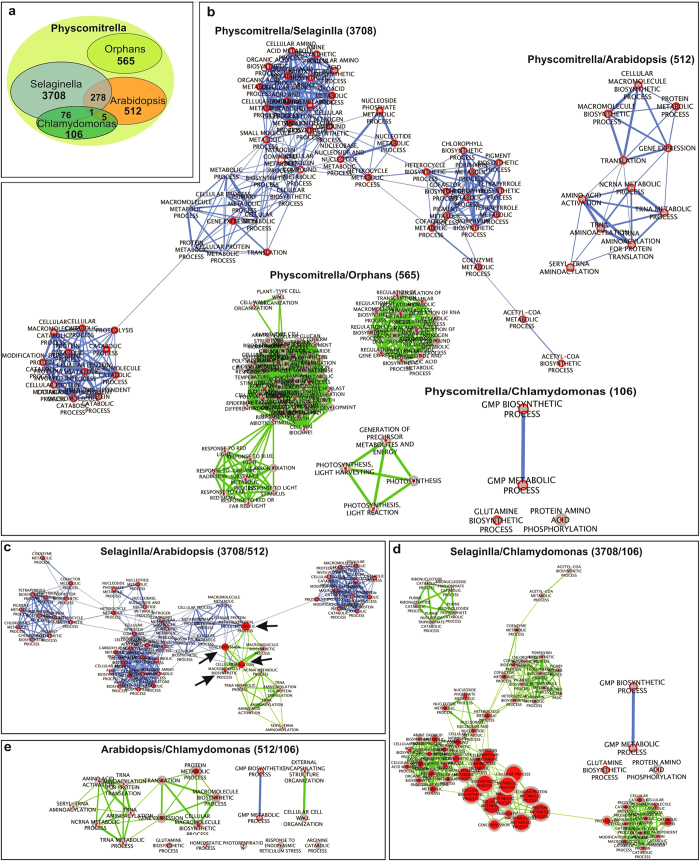
Evolutionary dynamics and orphan transcripts of DEGs. (**a**) Venn diagram showing overlap of *P. patens* DEGs between *C. reinhardtii*, *S. moellendorffii* and *A. thaliana* based on BLAST-P analysis as well as the *P. patens* orphan DEGs. (**b**) Differentiating the GO enriched functional categories of *P. patens* orphan DEGs with *P. patens*/*C. reinhardtii*, *P. patens*/*S. moellendorffii*, and *P. patens*/*A. thaliana* genes (green represents the orphans and blue represents the genes conserved with other model organisms). (**c**) Comparative analysis between GO enriched functional categories of *P. patens*/*S. moellendorffii* and *P. patens*/*A. thaliana* groups (green represents the *P. patens*/*A. thaliana* enriched functional groups and blue represents the *P. patens*/*S. moellendorffii* enriched functional groups, arrows indicate the shared and connected GO enriched functional categories between the two groups). (**d**) Comparative analysis between GO enriched functional categories of *P. patens*/*C. reinhardtii*, and *P. patens*/*S. moellendorffii* groups (green represents the *P. patens*/*S. moellendorffii* enriched functional groups and blue represents the *P. patens*/*C. reinhardtii* enriched functional groups). (**e**) Comparative analysis between GO enriched functional categories of *P. patens*/*C. reinhardtii*, and *P. patens*/*A. thaliana* groups (green represents the *P. patens*/*A. thaliana* enriched functional groups and blue represents the *P. patens*/*C. reinhardtii* enriched functional groups). Cytoscape and Enrichment Map was used for visualization of the GSEA results from BiNGO plug-in. Node size represent the number of genes in *P. patens*. The color varies based on the BiNGO *p-value* significance. Edge size reflects the number of overlapping genes between the two connected gene-sets.

Given the phylogenetic position of *P. patens*, the specific adaptations to the new environmental conditions required for the transition from aquatic to terrestrial life can be studied within this model plant^1^. To investigate the evolutionary conservation of stress responses in land plants, we conducted a comparative analysis of stressed-DEGs between unicellular algae (*C. reinhardtii*), bryophytes (*P. patens*), lycophytes (*S. moellendorffii*) and angiosperms (*A. thaliana*) to uncover the core networks of processes that led to the diversity of responses observed among extant plants. The resulting non-redundant 9,668 *P. patens* stressed-DEGs (Supplementary Dataset 6) were subjected to BLAST-P analysis (Supplementary Dataset 17), and 512, 3,708 and 106 genes were shared with *A. thaliana*, *S. moellendorffii*, and *C. reinhardtii*, respectively. Additionally, 565 genes were predicted to be orphan genes (Fig. 6a, Supplementary Datasets 18–20). Gene set enrichment analysis, in conjunction with ortholog analysis was used to identify enrichment, conservation, and rewiring of functional categories of the ortholog genes between *P. patens* and *A. thaliana*, *S. moellendorffii* and *C. reinhardtii.* The entire ortholog sets found between the aforementioned species were used to examine the functional categories. We identified the multiple GO enriched functional categories that were conserved or varied between these model organisms (Fig. 6b–e and Supplementary Datasets 22–25). We also compared the GO enriched categories of *P. patens* orphan genes with *P. patens*/*C. reinhardtii*, *P. patens*/*S. moellendorffii*, and *P. patens*/*A. thaliana* genes. Remarkably, we found that there is no shared GO enriched term in any functional group between the conserved and the orphan genes (Fig. 6b). A comparative analysis between GO enriched functional categories of *P. patens*/*C. reinhardtii*, *P. patens*/*S. moellendorffii*, and *P. patens*/*A. thaliana* genes indicated that the GO enriched genes for GMP (guanosine monophosphate) biosynthetic and GMP metabolic process among the *P. patens*/*C. reinhardtii* group are not connected with those from *P. patens*/*S. moellendorffii*, and *P. patens*/*A. thaliana* (Fig. 6d,e). On the other hand, the GO terms enriched with gene expression, translation and protein metabolic process are shared and connected between *P. patens*/*S. moellendorffii*, and *P. patens*/*A. thalina* groups (Fig. 6b). The comparison of *P. patens* stress regulated genes with unicellular algae, vascular and flowering plants revealed genomic changes concomitant with the evolutionary movement to land, including a general gene family complexity and loss of genes associated with different functional groups.

### Orphan genes of abiotic stress responses

Genes with no trans-species similarity (orphans) appear in all sequenced genomes. Several orphans have been implicated to play a role in plant responses to the environment and in lineage-specific traits; and some orphans are functional when introduced into evolutionarily distant species^30^. Improving our understanding of the origins of lineage-specific genes is key to gaining insights on how novel genes can arise and acquire functionality in different lineages^30^. The presence of lineage-specific genes was a striking and dominant feature revealed in the re-annotation of the *P. patens* genome (v1.6)^2^. In total, 565 of identified abiotic stress responsive genes in *P. patens* had no orthologs in other Viridiplantae (Fig. 6a,b), and they were present in the early and late stress responses (Supplementary Dataset 18). Generally, they appeared to be differentially regulated across all the stress treatments, but some genes were differentially regulated in a specific stress treatment. For example, Pp1s120_56V6.1 has no available GO annotation but appears to be regulated only in ABA 4.0 h, and Pp1s124_18V6.1 encodes anthocyanin transcriptional regulator and is regulated only in ABA 0.5 h (Supplementary Dataset 18). The hypothetical protein (Pp1s27_173V6.1) appears to be up regulated only in salt 0.5 h and drought treatments (Supplementary Dataset 18).

**Figure 7:**
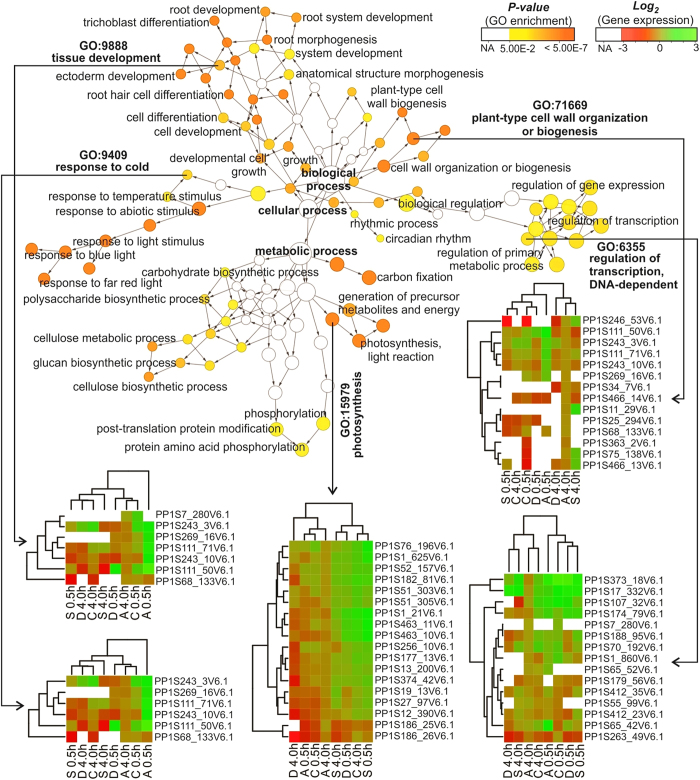
A network of GO categories combined with representative heatmaps for *P. patens* orphan stressed-DEGs. BiNGO, a cytoscape plugin was used to visualize the GO terms that were statistically (hypergeometric test) over-represented from the *P. patens* orphan DEGs. Representative heatmaps clustering analysis of gene expression based on RPKM log_2_ ratio. The color scale (from white, yellow to dark orange) is based on (corrected) *p-value* (5% FDR, p = 0.05). Dark orange categories are most significantly overrepresented, white nodes are not significantly overrepresented (NA); they are included to show other nodes in the context of the GO hierarchy. The area of a node is proportional to the number of genes in the test set annotated to the corresponding GO category. The heatmaps were generated using the R gplots package. The RPKMs were used as input for the hierarchical clustering using Euclidean measure to obtain distance matrix, complete agglomeration and dendograms. The color scale (from white, red to green) is based on log_2_ fold change. Green represents the up-regulated genes; red represent the down-regulated ones among the different stress treatments. White color indicated no gene expression in certain stress treatment (NA), A: ABA, C: cold: D: drought, S: salt.

Over-representation of GO terms combined with differentially regulated orphan genes across all the stress treatments were determined by using the Biological Networks Gene Ontology tool (BiNGO). These were categorized into 82 functional groups (Supplementary Dataset 22). Most of these groups showed statistically significant GO representation (Fig. 7 and Supplementary Fig. S5). Among these groups, the terms of response to cold (GO:0009409), response to abiotic stimulus (GO:0009628), response to temperature stimulus (GO:0009266) and response to stimulus (GO:0050896) showed significant over-representation based on the obtained *P-values* (Supplementary Fig. S5). Notably, over-representation of GO terms in *P. patens* orphan stressed-DEGs was observed for different functional groups involved in different morphological, physiological and metabolic pathways. This suggests that there are considerable differences between the physiological processes among these genes.

## Discussion

The *P. patens* is an important model organism for *evo*-*devo* studies. During 470 million years of divergent evolution, extant mosses and seed plants developed different strategies for survival, leading to evolution of the dominant gametophytic and sporophytic, respectively^1^,^31^. Were the evolution of these morphological changes accompanied by changes and innovation in new stress responses? One of the main objectives of evolutionary conservation analysis between bryophytes, algae and angiosperms is to uncover the core networks of processes that led to the diversity of responses observed among extant plants. By comparative analysis of gene function in the species representing different evolutionary steps, it is possible to differentiate between gene families that emerged recently in the course of evolution and conserved gene families encoding proteins with fundamental functions^3^. Our data are discussed in an evolutionary context in order to dissect plant response strategies to stress, also to reveal stress induced expression of moss-specific genes, which may have contributed to the evolutionary success and survival of these poikilohydric plants.

Adaptation to land also required the evolution of proteins that protect against stresses. One example of this is the expansion of the heat shock protein 70 (HSP70) family to 13 cytosolic members in *P. patens*, whereas all algal genomes sequenced to date encode one single cytosolic HSP70. Our results indicated that these members are present and expressed among all the stress treatments with different expression levels. Some were highly expressed like: Pp1s2_216V6.1, Pp1s351_21V6.1 and Pp1s97_279V6.1, but others like: Pp1s351_24V6.1, Pp1s262_62V6.1and Pp1s298_26V6.1, were expressed at a low level (Supplementary Dataset 5).

Dehydration tolerance in seeds is dependent on ABA to induce expression of seed-specific genes, such as late embryogenesis abundant proteins (LEAs), a group of proteins that accumulate during dehydration. *P. patens* contain orthologs of LEA genes and other genes expressed during the abiotic stress responses. The *P. patens* genome contains putative homologs of the *A. thaliana* ABA receptors, such as, the transcription factor ABI5, which implicates it in the regulation of ABA-mediated gene expression.

Our analysis identified that ABA- and drought- responsive genes include those encoding a number of LEA proteins and a dehydration-responsive element-binding protein (DREB) transcription factor. In *A. thaliana*, some dehydration-responsive genes are activated by transcription factors mediating an ABA-dependent pathway, which include basic-leucine zipper (bZIPs), MYC/MYB and ABI3/VPI families that recognize specific binding sites in the promoter regions of these genes^32^. Similarly in *P. patens* ABA-specific induction operates via binding of bZIP transcription factors to ABREs^33^. Salt-induced genes have been identified in *P. patens* encoding regulatory proteins such as calmodulin binding proteins^34^, a Ca^2+^-ATPase^35^, and the AP2/EREBP transcription factor PpDBF1^36^. In agreement with our analysis, a high number of salt-specifically expressed genes have been identified and a large number of salt-DEGs were expressed after 0.5 and 4.0 h exposure to 350 mM NaCl, including the aforementioned genes. In plants, once primary sensors perceive a low-temperature signal, Ca^2+^ channels are activated and cytosolic levels of Ca^2+^ increase triggering the activation of proteins which provide cellular protection such as the activation of the LEA family genes and the expression of cold-regulated genes (COR)^37^. The activation of the related signal transduction pathway will result in the accumulation of osmoprotectants like sugars, amines and compatible solutes which eventually lead to membrane stabilization and altered gene expression to provide protection at all levels^38^. Our results indicated ABA-responsive genes in *P. patens* were induced when treated with salt and cold, which suggests the involvement of these genes in response to multiple stresses. Such cross-talk can be mediated by calcium, a second messenger, which increases during the various abiotic stresses and induces the signaling pathway and consequently the expression of various genes that have a role in maintaining the cell homeostasis.

Orphans may be defined as genes with coding sequences utterly unique to the species; in other words, genes that presumably produce previously novel proteins. However, every evolutionary lineage harbors orphan genes that lack homologues in other lineages and whose evolutionary origin is poorly understood^30^. Considering the high number of orphan genes among the persistent stressed-DEGs (Fig. 6a,b and Fig. 7) we conclude an important role of species- or lineage- specific genes in the acquisition of abiotic stress tolerance in mosses. Species- or lineage- specific genes could also be responsible for poikilohydry, as this phenomena is also noted in many forms of algae.

Because the majority of DEGs representing these processes have orthologs in other Viridiplantae, we conclude that basic molecular mechanisms of abiotic stress responses may occur in the gametophyte of mosses (the dominant form in bryophytes) and the sporophyte of flowering plants (the dominant form in vascular plants), and this will extend our understanding of stress molecular biology and provide a foundation for future studies on the stress regulatory mechanisms of *P. patens* and other plant species. Additionally, Species or lineage-specific stress-regulated genes not found in higher plants suggest that these unknown and un-classified transcripts might represent valuable targets for crop plant improvement in a changing climate via targeted gene replacement.

## Methods

### Plant materials and stress treatments

The cultivation of *P. patens* was performed in liquid mineral medium [250 mg l^−1^ KH_2_PO_4_, 250 mg l^−1^ MgSO_4_.7H_2_O, 250 mgl^−1^ KCl, 1000 mg l^−1^ Ca(NO_3_)_2_.4H_2_O, 12.5 mg l^−1^ FeSO_4_.7H_2_O, pH 5.8 with KOH]^39^. Freshly disrupted protonemata were sub-cultured for 5 days and transferred to fresh medium and cultured over night in Erlenmeyer flasks on shakers at 125 rpm. For salt and ABA treatments the medium was supplemented with 350 mM NaCl while for ABA treatments, *cis-trans* ABA was added to a final concentration of 10^−5^ M. For drought treatments, the protonemata tissues form Erlenmeyer flasks were transferred directly to the plastic base of a 9 cm Petri dishes. The dishes (uncovered) were placed in transparent plastic containers containing saturated NaCl solution in order to maintain the atmosphere of relative humidity^9^. Cold treatments were performed by transferring Erlenmeyer flasks of the protonemata tissues to the fridge at 4 °C. During treatment, the temperature of the medium was 4 °C. The control plants were grown in parallel and harvested at the same time as the abiotic stress treated samples. After 0.5 and 4 hours moss material of three flasks per treatment were harvested, pooled and frozen in liquid nitrogen.

### RNAseq libraries construction and sequencing

Total RNA was isolated from protonema tissues using TRIzol reagent (Invitrogen, USA) following the manufacturer’s instructions. The *P. patens* RNAseq libraries were prepared using TruSeq RNA sample prep kit (Illumina, Inc.) following the manufacturer’s instructions. Briefly, TruSeq RNA sample prep kit converts the poly-A containing mRNA in total RNA into a cDNA library using poly-T oligo-attached magnetic bead selection. Following mRNA purification, the RNA is chemically fragmented prior to reverse transcription and cDNA generation. The fragmentation step results in an RNAseq library that includes inserts that range in size from approximately 100–400 bp. The average insert size in an Illumina TruSeq RNA sequencing library is approximately 200 bp. The cDNA fragments then go through an end repair process, the addition of a single ‘A’ base to the 3’ end and then ligation of the adapters. Then, the products are purified and enriched with PCR to create the final double stranded cDNA libraries. Finally, libraries quality control and quantification were performed with a Bioanalyzer Chip DNA 1000 series II (Agilent) and sequenced directly using the high-throughput Illumina HiSeq sequencing system (Illumina, Inc.). The raw reads for each library were deposited in NCBI BioSample database and they are accessible through Sequence Read Archive (SRA) accession number SRP050162.

### Alignment and analysis of Illumina reads against *P. patens* reference genome

Paired End (PE) reads from each library (four abiotic stress treatments with selected time points 0.5 and 4.0 hours in addition to the control sample) were processed using CASAVA version 1.8.2 package in Fastq format. Trimmomatic package^40^ was used to remove adapters and filter out low quality bases (< Q20), and only those reads which showed quality score of 20 or higher were retained. Filtered sequences reads were mapped to the *P. patens* genome V1.6^2^ (Cosmoss database; http://cosmoss.org/, data can also be found in JGI (Phytozome; http://www.phytozome.net/physcomitrella.php) by using a CLCbio Genomics workbench 6.1 (http://www.clcbio.com). The CLCbio Genomics workbench used uncompressed suffix array and smith-waterman algorithm for reference mapping.

### Global gene expression and differential gene expression analysis

A comparison of commonly and uniquely expressed genes across the treatments was done using the online tool Venny^41^. The comparison was done for control versus all stress treatments including two time points. Reads were mapped to the reference genome of *P. patens* (v1.6)^2^ to calculate normalized gene expression values using the RPKM metrics^19^. In order to filter out sequencing and mapping artifacts, we chose RPKM values of ≥10 as our threshold. The differential expression is calculated for each gene by using the log_2_-ratio calculation; differential expression is reported as the log_2_-ratio of two expressed values. Equal expression values for each gene would have a log_2_-ratio of zero, while the genes are up-regulated if this ratio is above zero and down-regulated if the ratio is below zero, a log ratio of 1 represents a 2-fold change^42^. The gene expression profiling was done using Tophat^43^, Cufflinks^20^ and CummeRbund package (http://compbio.mit.edu/cummeRbund/). The heatmaps and dendrogram are generated using R package (http://www.r-project.org/) to compare the expression profiling of the transcriptome in different stress treatments. Hierarchical clustering was performed using Pearson’s correlation coefficient as distance metric and the average agglomeration method^23^. Clustering dendrograms were examined below the 0.15 height threshold, allowing a close inspection of genes clustered at or above a cluster-average correlation coefficient of 0.91.

### Quantitative real-time PCR (qPCR)

For qPCR, cDNA corresponding to 50 ng of total RNA was used per transcript to be quantified. Quantitative PCR reactions were performed on Applied Biosystems StepOnePlus instrument system using KAPA SYBR FAST One-Step qRT-PCR Kit (Kapa Biosystems, USA) with gene-specific primers according to the manufacturer’s instructions. Data were normalized relative to Pp1s13_134V6.1 and Pp1s56_240V6.1 genes, which exhibited relatively, stable expression levels in all abiotic stress treatments and in the control sample as well. Melting curves were analyzed on the product to determine if only a single product was amplified without primer dimers and other bands; melting curve analysis was performed for each primer pair before further analyses. Relative quantitative analysis was performed by comparative quantitation using StepOne v2.3 software. All reactions were run in triplicate. The primers for subsequent qPCR reactions are listed in Supplementary Table S5.

### Gene ontology and gene set enrichment analysis

For the DEGs, the GO^44^ annotation of the recent *P. patens* genome annotation (v1.6)^2^ was analyzed with Blast2GO (version 2.3.5) (http://www.blast2go.org/) with the default parameters^45^. To assess the GSEA^46^ for the DEGs we identified, we used the public web tool g:Profiler (http://biit.cs.ut.ee/gprofiler)^24^ for functional characterization of the gene lists. g:Profiler comprises of five components (g:GOSt for gene group functional profiling, g:Cocoa for compact comparison of annotations, g:Convert for gene ID conversions, g:Sorter for expression similarity searches and g:Orth for orthology searches). g:GOSt was used to identify functional characterization of the DEG lists. The reference GO annotation sets are retrieved from the Ensembl database (Release 75) and Ensembl Genomes (Release 22) and classified into three components: biological process, molecular function and cellular component. Fisher’s one-tailed test (cumulative hypergeometric probability) was used to calculated *P-value* and Benjamini-Hochberg False Discovery rate with the cut-off value after correction <0.05 was used for multiple testing correction. The GO terms that have *P-values* below the threshold of <0.05 were considered as statistically significant. The level plot for selected GO terms was done under R suite software (http://www.r-project.org/). g:Profiler also provides the ortholog mapping (g:Orth Ortholgoy search). The input gene IDs were automatically converted via Ensemble gene IDs using g:Convert and then used for ortholog mapping based on Ensemble alignments.

### Evolutionary dynamics and orphan transcripts analysis

A detailed BLAST analysis of *P. Patens* DEGs against all organisms in the taxonomic group of Viridiplantae was carried out to define the evolutionary conservation of these genes within this lineage. For this purpose the entire available proteins sequences of Viridiplantae were downloaded from UniProt database (http://www.uniprot.org/). A BLAST database was created with these sequences. The protein sequences of 9,668 DEGs of *P. Patens*, extracted from *P. Patens* protein sequences (Cosmoss database; http://cosmoss.org/) were used as queries against the Viridiplantae BLAST database and were searched for homology at the *E-*values of 0.01 or lower. For the purposes of this study, we used *C. reinhardtii*, *S. moellendorffii* and *A. thalina* proteomes for comparative analysese; if no homology was found across any of Viridiplantae species in our database, we considered the query as an orphan gene.

GSEA was used to identify enrichment, the conservation or rewiring of functional categories of the orthologue genes that had a common ancestor between *P. Patens* to *C. reinhardtii*, *S. moellendorffii* and *A. thalina*. Over-representation of GO terms in a set of genes was determined by using the Biological Networks Gene Ontology tool (BiNGO)^47^. BiNGO retrieved the relevant GO annotation then tested for significance using the Hypergeometric test and corrected multiple testing using Benjamini and Hochberg false discovery rate (FDR) correction. The Jaccard coefficient was used to compare the similarity between enrichment sets A and B. It was defined as the intersection between group A and B divided by their union, the results from BiNGO were then visualized through the enrichment map. Cytoscape and enrichment map was used for visualization of the GSEA results from the BiNGO plug-in.

## Additional Information

**How to cite this article**: Khraiwesh, B. *et al.* Genome-wide expression analysis offers new insights into the origin and evolution of *Physcomitrella patens* stress response. *Sci. Rep.* **5**, 17434; doi: 10.1038/srep17434 (2015).

## Supplementary Material

Supplementary Tables and Figures

Supplementary Datasets 1-25
