# Supplementary material for: Genome-wide expression analysis offers new insights into the origin and evolution of Physcomitrella patens stress response
Source: Sci Rep. 2015 Nov 30;5:17434. doi: 10.1038/srep17434 (PMC4663497; doi:10.1038/srep17434)
Supplement: Supplementary Tables and Figures [file srep17434-s1.pdf]

Supplementary information

Genome-wide expression analysis offers new insights into the origin and evolution of *Physcomitrella patens* stress response

Basel Khraiwesh[1,2,3*], Enas Qudeimat[2,3§], Manjula Thimma[3§], Amphun Chaiboonchoe[1§], Kenan Jijakli[1], Amnah Alzahmi[1], Marc Arnoux[2], Kourosh Salehi-Ashtiani[1,2*]

[1]Laboratory of Algal, Systems, and Synthetic Biology, Division of Science and Math, New York University Abu Dhabi, Abu Dhabi, UAE
[2]Center for Genomics and Systems Biology, New York University Abu Dhabi, Abu Dhabi, UAE
[3]Division of Biological and Environmental Sciences and Engineering, King Abdullah University of Science and Technology, Thuwal, KSA

§ These authors contributed equally to this work.
* Corresponding Authors: Basel Khraiwesh (bhk1@nyu.edu) and Kourosh Salehi-Ashtiani (ksa3@nyu.edu), Center for Genomics and Systems Biology, New York University Abu Dhabi, Abu Dhabi, UAE

Supplementary Tables

Table S1: **Summary of reads and expressed genes based on the RNASeq data from *P. patens* abiotic stress treatments**.

Features	0.5 h				4.0 h				
	ABA	Cold	Drought	Salt	ABA	Cold	Drought	Salt	Control
**Total Reads**	24,572,598	25,701,402	22,727,146	21,066,004	23,995,124	26,269,396	24,271,618	22,921,412	26,827,908
**Mapped Reads**	21,965,889	22,735,394	19,806,506	18,141,685	21,455,483	23,039,577	21,163,981	19,818,724	23,929,288
	89.39%	88.46%	87.15%	86.12%	89.42%	87.71%	87.20%	86.46%	89.17%
**Uniquely Mapped Reads**	20,512,728	21,173,562	19,013,472	16,951,836	19,917,780	22,061,344	20,286,906	18,473,814	22,601,958
	83.48%	82.38%	83.66%	80.47%	83.01%	83.98%	83.58%	80.60%	84.20%
**Non-Uniquely Mapped Reads**	1,453,161	1,561,832	793,034	1,189,849	1,537,703	978,233	877,075	1,344,910	1,327,330
	5.91%	6.08%	3.49%	5.65%	6.41%	3.72%	3.61%	5.87%	4.90%
**Unmapped Reads**	2,606,709	2,966,008	2,920,640	2,924,319	2,539,641	3,229,819	3,107,637	3,102,688	2,898,620
	10.61%	11.54%	12.85%	13.88%	10.58%	12.29%	12.80%	13.54%	10.80%
**Number of Expressed Genes**	19,591	20,003	19,438	19,896	19,575	19,909	19,312	19,489	19,822

Table S2: **The table shows up-regulated genes at 0.5 h with fold change > 50 in RPKM**. This table is in support with Fig. 4a.

**Gene ID**	**ABA 0.5 h**	**Cold 0.5 h**	**Drought 0.5 h**	**Salt 0.5 h**	**Description**
**Pp1s370_29V6.1**	73.06521739	58.34782609	658.8695652	1117.543478	Late embryogenesis abundant protein, LEA-3
**Pp1s2_226V6.1**	57.07692308	63.74358974	400.7179487	590.025641	F15D2.42; stress-responsive protein, putative [*Arabidopsis thaliana*]
**Pp1s37_28V6.1**	68.85	53.075	69.175	69.8	Ubiquitin ligase protein
**Pp1s266_43V6.1**	109.4285714	58.28571429	418.2857143	423.1428571	F11F8.28; hydrolase, alpha/beta fold family protein [*Arabidopsis thaliana*]
**Pp1s276_3V6.1**	94.48275862	19.89655172	189	156.5517241	Transcriptional factor
**Pp1s55_253V6.1**	128.8571429	54.57142857	937.2857143	150.8571429	Transcriptional factor
**Pp1s43_12V6.1**	141.5625	235.5625	187.1875	82.3125	Sigma factor sigb regulation protein

Table S3: **The table shows down-regulated genes at 0.5 h with fold change >10 in RPKM**. This table is in support with Fig. 4b.

Gene ID	ABA 0.5 h	Cold 0.5 h	Drought 0.5 h	Salt 0.5 h	Description
**Pp1s660_1V6.1**	-4.439415589	-3.324242282	-3.920464095	-3.939613353	Non-symbiotic hemoglobin class 1
**Pp1s2_7V6.1**	-6.631540867	-4.561151539	-8.14611404	-5.631540867	Zinc finger, B-box;CCT domain
**Pp1s251_59V6.1**	-6.808964175	-4.860596943	-9.001609253	-4.539837496	Beta-expansin 3
**Pp1s11_29V6.1**	-3.459431619	-3.392317423	-4.409526713	-3.738686377	Expansin 2

Table S4: **The table shows up and down-regulated genes at 0.5 h with similar expression trend**. This table is in support with Fig. 4c.

Gene ID	ABA 0.5 h	Cold 0.5 h	Drought 0.5 h	Salt 0.5 h	Description
**Pp1s100_117V6.1**	1.347084708	1.285478548	1.355885589	1.184268427	ATP synthase subunit beta
**Pp1s122_140V6.1**	1.523911875	1.276732939	1.4191295	1.19398173	T10P11.12; seven transmembrane MLO family protein / MLO-like protein 1 (MLO1) [*Arabidopsis thaliana*]
**Pp1s143_71V6.1**	0.629011553	0.740693196	0.983311938	0.836970475	DS545032.1; 487504; protein_coding
**Pp1s185_2V6.1**	0.66713593	0.708431097	0.86305334	0.763882371	Glutaredoxin c4
**Pp1s36_294V6.1**	1.042935417	1.027845145	1.233450103	1.118835893	Acyl-binding protein
**Pp1s37_172V6.1**	0.902439024	1.291666667	1.254065041	1.131097561	No description; protein_coding
**Pp1s39_347V6.1**	0.642169908	0.631832139	0.743398158	0.6209826	Proteasome epsilon chain precursor
**Pp1s60_311V6.1**	0.792592593	0.955555556	1.385185185	1.074074074	No description
**Pp1s73_34V6.1**	1.559370529	1.904148784	2.258941345	2.075822604	Multiple inositol polyphosphate phosphatase 1
**Pp1s84_242V6.1**	1.322714379	1.343661357	1.635391665	1.642592189	No description

Table S5: **The primer sequences of the randomly selected and internal reference genes used for quantitative real-time PCR (qPCR) analysis.** This table is in support with Fig. 5.

Gene ID	Forward Primer	Reverse Primer	Gene Annotation
**Pp1s13_134V6.1**	CATGATTGATCGCTTGTTGG	ACCGCGATCTTTATCACCTG	3-hydroxyisobutyryl-coenzyme A
**Pp1s56_240V6.1**	AGTGCTGGGTTTCATTCGAC	AGCCATGTTACATCCGGAAA	Riboflavin kinase
**Pp1s1722_1V6.1**	AGAGAGTGCGCAGACTGGAT	ACCCGGATTCAACCAAACTT	mRNA sequence for carbonic anhydrase
**Pp1s52_261V6.1**	ATGTTGCACGGTCACAAGAA	CGAGGAAGCCCTTCTTCTCT	Polyadenylation Signal (PAS)
**Pp1s27_129V6.1**	AGAACACAGAGGCCAGCAGT	ATCGTCATGTCGTTCCCATT	No description
**Pp1s274_60V6.1**	GGCGAGCTTAGGTACTGCAC	CTTGGAGGTGACGACCTGAT	F4F15.180; expressed protein [*Arabidopsis thaliana*]
**Pp1s49_268V6.1**	TTCAGGAGGGCTGTGGATAA	AGATCCCAAATGAGCTGCAC	No description
**Pp1s4821_1V6.1**	CGAGCACACAATCATGAACC	CTCCTTCCTCAAACAGGAACC	mRNA sequence for carbonate dehydratase
**Pp1s370_29V6.1**	AAGGATGCGACCTTGAACAC	AAACCCTGCAGTGTGTTTCC	Late embryogenesis abundant protein, LEA-3
**Pp1s55_253V6.1**	TCTGGGGATGAAGACTCAGG	TTCCACCTTAACCCATTCCA	Transcriptional factor
**Pp1s2_7V6.1**	GAAACCGAAGGGAGAGAAGG	CCTATCGGACCAGGCTGTAA	Zinc finger, B-box;CCT domain
**Pp1s251_59V6.1**	GTGATCCCAGATTGGTTTGG	CTTCGTAAACTCCGCCTCTG	Beta-expansin 3

Supplementary Figures

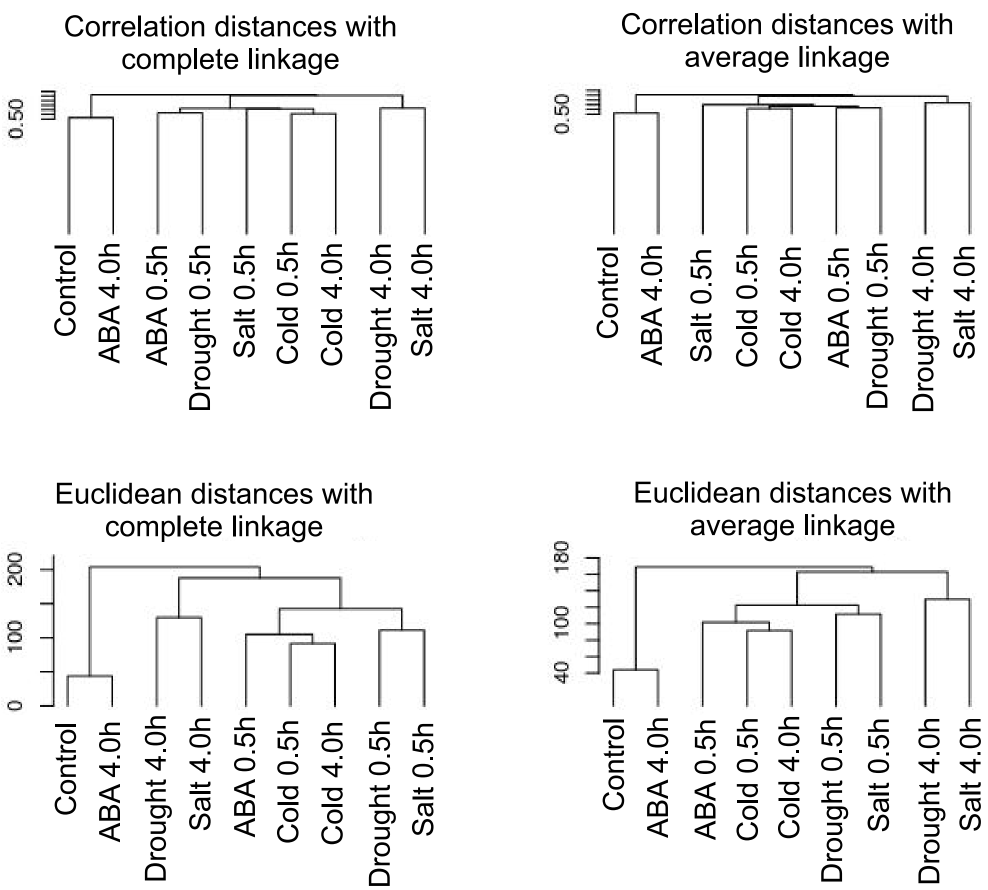

Figure S1: Dendrograms describing the expressions profile similarities between stress responses. The dendrograms are generated using gplots package in R (https://cran.r-project.org/web/packages/gplots/index.html). Each dendrogram is shown with distance and clustering methods in their titles respectively.

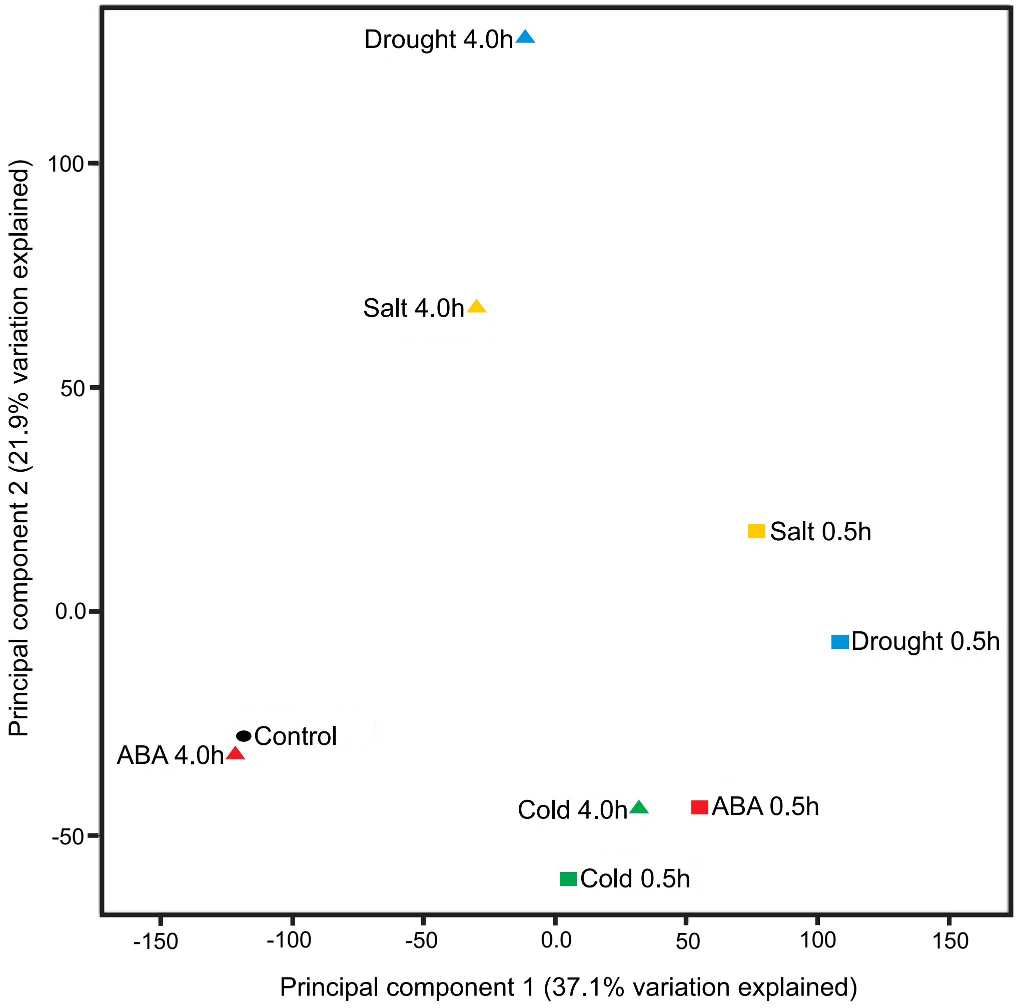

Figure S2: The Principal Component Analysis (PCA) plot as shown above depicts the grouping of expression profiles of each sample in the experiment. This plot has been generated using R programming language (https://www.r-project.org/) with default parameters.

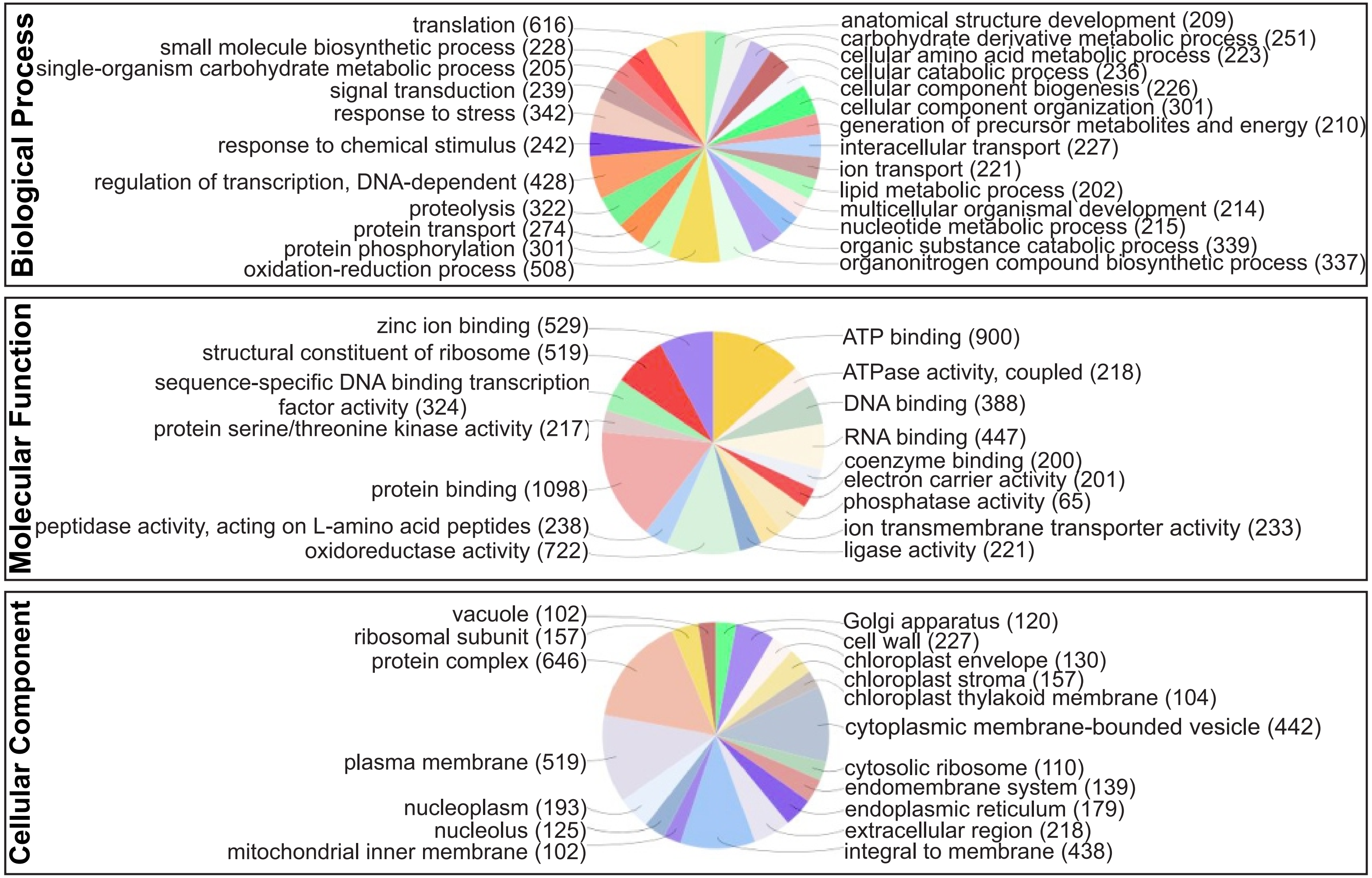

Figure S3: Pie chart representing Gene Ontology (GO) terms distribution in *P. patens* DEGs. DEGs were annotated in at least one of the three GO categories: biological process, molecular function and cellular component. Sequence distribution filtered by number of sequences for biological process, molecular function and cellular component with cutoff 200, 200, and 100, respectively. See **Supplementary Datasets 12-14** for the complete lists.

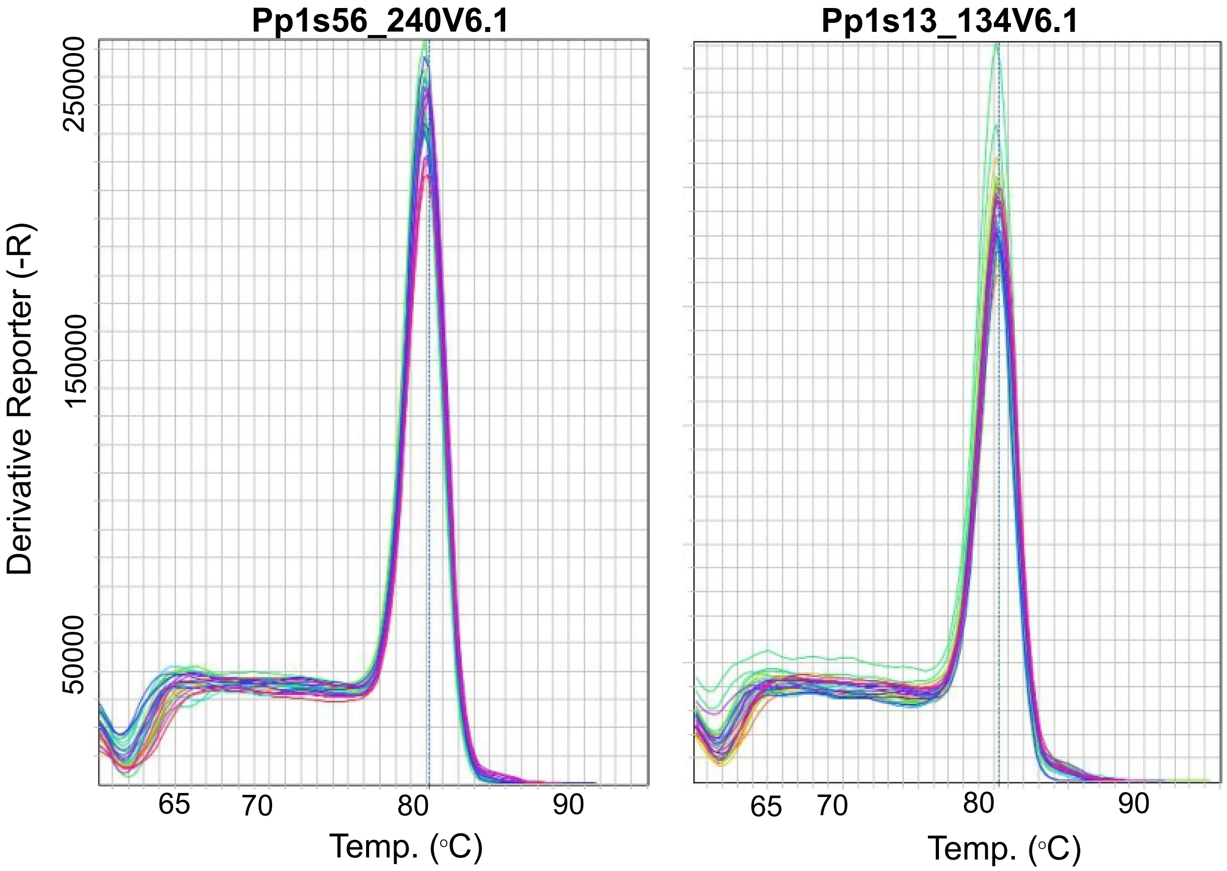

Figure S4: Melting curves analyses for the two reference genes were used for normalization of DEGs qPCR data.

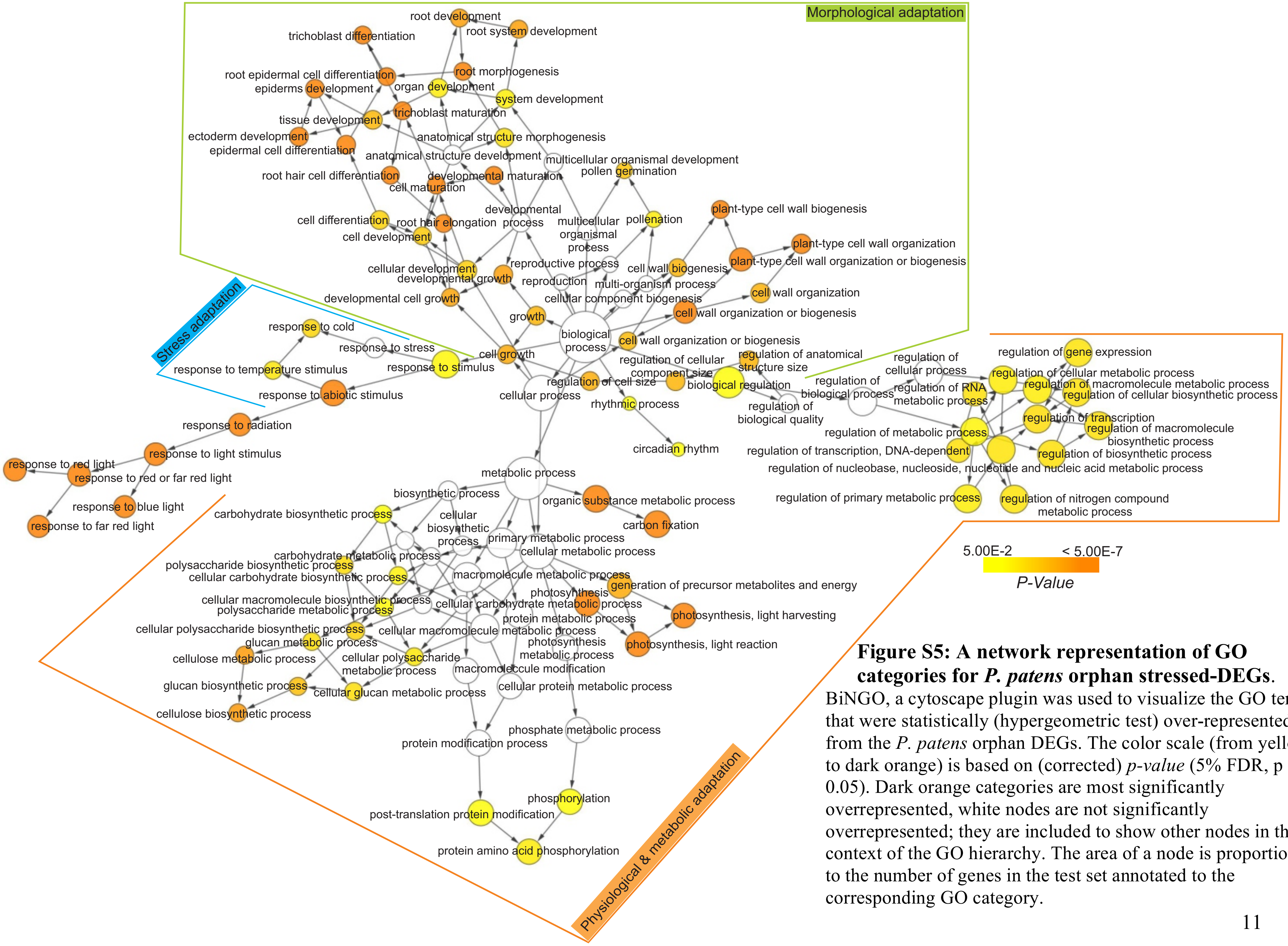

Figure S5: A network representation of GO categories for *P. patens* orphan stressed-DEGs. BiNGO, a cytoscape plugin was used to visualize the GO terms that were statistically (hypergeometric test) over-represented from the *P. patens* orphan DEGs. The color scale (from yellow to dark orange) is based on (corrected) *p-value* (5% FDR, p = 0.05). Dark orange categories are most significantly overrepresented, white nodes are not significantly overrepresented; they are included to show other nodes in the context of the GO hierarchy. The area of a node is proportional to the number of genes in the test set annotated to the corresponding GO category.

Supplementary Datasets 1-25

Supplementary Dataset 1: List of genes representing the degree of overlap among gene expression of *P. patens* control and ABA treated samples at different time points.

Supplementary Dataset 2: List of genes representing the degree of overlap among gene expression of *P. patens* control and cold treated samples at different time points.

Supplementary Dataset 3: List of genes representing the degree of overlap among gene expression of *P. patens* control and drought treated samples at different time points.

Supplementary Dataset 4: List of genes representing the degree of overlap among gene expression of *P. patens* control and salt treated samples at different time points.

Supplementary Dataset 5: List of expressed genes based on RPKM value across all abiotic stress treatments and the control sample. GO terms (inferred by BLAST2GO) based on the *P. patens* V1.6.

Supplementary Dataset 6: List of DGEs of *P. patens* in response to ABA, cold, drought and salt treatment at two different time points.

Supplementary Dataset 7: List of DEGs representing the degree of overlap among the two time points across all abiotic stress treatments.

Supplementary Dataset 8: List of up regulated genes of *P. patens* representing the degree of overlap among all studied stress treatments at 0.5 an hour.

Supplementary Dataset 9: List of up regulated genes of *P. patens* representing the degree of overlap among all studied stress treatments at 4.0 hours.

Supplementary Dataset 10: List of down regulated genes of *P. patens* representing the degree of overlap among all studied stress treatments at 0.5 an hour.

Supplementary Dataset 11: List of down regulated genes of *P. patens* representing the degree of overlap among all studied stress treatments at 4.0 hours.

Supplementary Datasets 12-14: GO terms (inferred by BLAST2GO) for DEGs based on the *P. patens* V1.6 GO annotation. Biological process (**Supplementary Dataset 12**), molecular function (**Supplementary Dataset 13**), cellular component (**Supplementary Dataset 14**).

Supplementary Dataset 15: List of enriched functional groups for up regulated genes based on GSEA.

Supplementary Dataset 16: List of enriched functional groups for down regulated genes based on GSEA.

Supplementary Dataset 17: A detailed BLAST-P analysis and hits of *P. patens* stressed-DEGs against all organisms in the taxonomic group of Viridiplantae.

Supplementary Datasets 18-21: List of DEGs representing the degree of overlap for evolutionary dynamics and orphan transcripts integrity. Orphan genes (**Supplementary Dataset 18**), *P. patens*/*C. reinhardtii* (**Supplementary Dataset 19**), *P. patens*/*S. moellendorffii* (**Supplementary Dataset 20**), *P. patens*/*A. thaliana* (**Supplementary Dataset 21**).

Supplementary Datasets 22-25: GO enrichment analysis of DEGs associated with evolutionary dynamics for *P. patens* orphans (**Supplementary Dataset 22**), *P. patens*/*C. reinhardtii* (**Supplementary Dataset 23**), *P. patens*/*S. moellendorffii* (**Supplementary Dataset 24**) and *P. patens*/*A. thaliana* genes (**Supplementary Dataset 25**).
